# Two conjectures on 3D Voronoi structures: a toolkit with biomedical case studies

**DOI:** 10.1039/d4me00036f

**Published:** 2024-05-24

**Authors:** Lucy Todd, Matthew H. W. Chin, Marc-Olivier Coppens

**Affiliations:** a Centre for Nature Inspired Engineering & Department of Chemical Engineering, University College London Torrington Place London WC1E 7JE United Kingdom m.coppens@ucl.ac.uk

## Abstract

3D Voronoi scaffolds are widely applied in the field of additive manufacturing as they are known for their light weight structural resilience and share many topological similarities to various natural (bone, tumours, lymph node) and synthetic environments (foam, functionally gradient porous materials). Unfortunately, the structural design features that promote these topological similarities (such as the number of vertices) are often unpredictable and require the trial and error of varying design features to achieve the desired 3D Voronoi structure. This article provides a toolkit, consisting of equations, based on over 12 000 3D Voronoi structures. These equations allow design features, such as the number of generating points (*G*), to be efficiently and accurately predicted based on the desired structural parameters (within ±3*G*). Based on these equations we are proposing, to the best of our knowledge, two new mathematical conjectures that relate the number of vertices or edges, and the average edge length to *G* in Voronoi structures. These equations have been validated for a wide range of parameter values and Voronoi network sizes. A design code is provided allowing any of over 12 000 structures to be selected, easily adjusted based on user requirements, and 3D printed. Biomedical case studies relevant to T-cell culturing, bone scaffolds and kidney tumours are presented to illustrate the design code.

## Abstract 2

Voronoi structures can model a wide variety of environments, but their unique design requirements hamper their efficient creation. This work introduces four, accurate equations, including two new conjectures that greatly increase this efficiency.

## Graphical abstract

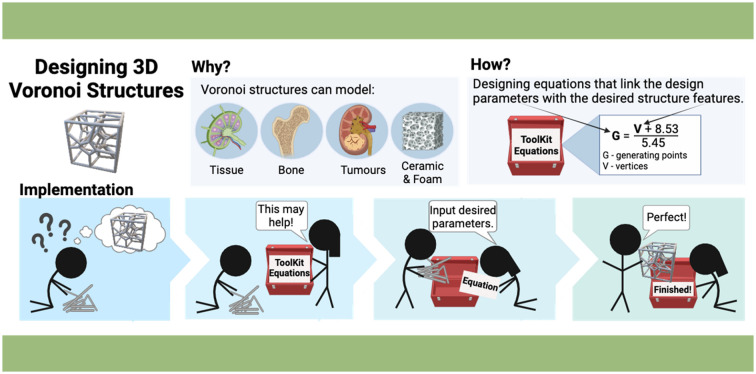

**Design, System, Application**

3D Voronoi structures are topologically and geometrically similar to a wide range of biological and engineering structures. These include bone, tissue, tumours, ceramics, and foams. Voronoi structures can be 3D printed to characterise them and test how guest confinement affects systems behaviour, such as the strength of a patient's bone or intracellular interactions. Voronoi structures can also be used to decrease the cost of printing solid objects. Instead of printing a solid plastic object, its shell could be printed with a Voronoi pattern housed inside to decrease ink use. However, Voronoi structures are currently constructed by defining the central points of each Voronoi cell, rather than the final nodes and edges that make up the structure. This is inefficient and complicates building highly specific 3D Voronoi structures. This article lists four equations that accurately predict the number of central points required to build a specific Voronoi structure based on the desired number of nodes, edges, average edge length and Euler's characteristic. We propose two mathematical conjectures that we found to apply to any of the shown Voronoi structures and might be general. This means highly specific 3D Voronoi structures can now be designed within seconds.

## 1 Introduction

Voronoi diagrams or tessellations (2D) and structures or networks (3D) are geometric arrangements that partition an allocated space into cells based on the proximity of pre-defined points. They have been widely studied and applied since their introduction in Western literature by René Descartes in 1644.^1^ Voronoi diagrams have been used to locate cholera outbreaks,^2^ select rain gauge locations,^3^ find galaxy clusters^4^ and model functionally gradient porous materials.^5^ Voronoi structures have been used to model foams,^6,7^ polycrystalline alumina,^8^ nanoporous heterogeneous materials,^9^ epithelial tissues,^10–12^ tumours,^13^ lymph node microenvironments^14,15^ and bone microenvironments.^16–18^ The emergence of additive manufacturing has extended the application of Voronoi tessellation from mere analysis to the generation of 3D scaffolds, *i.e.*, Voronoi structures with mesh wrapped around the wireframes. Voronoi scaffolds have been 3D printed as supports for hollow objects,^19^ interlocking architecture,^20^ optimal lightweight structures,^21–23^ patient specific bone scaffolds,^24,25^ for surgical practice,^26^ for patient education of upcoming procedures,^27^ regenerative medicine and wound healing techniques,^28^ implantable drug delivery^29^ and T-cell culturing environments.^14^

**Fig. 1:**
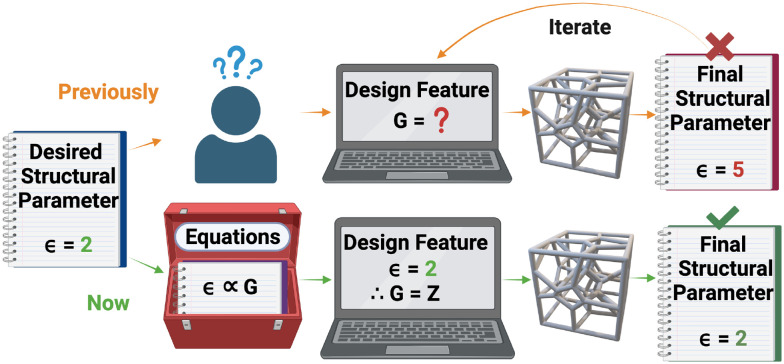
A graphical description of the purpose of this research: to find connections between the Voronoi design features and resulting structural parameters to efficiently forward engineer Voronoi structures. Average edge length and number of generating points are abbreviated to *ε* and *G*, respectively.

The wide application of 3D Voronoi structures in the biomedical, architectural and design fields stems from their light-weight abilities, customizable properties and the topological similarities they share with the various environments listed above. Voronoi structures are highly cost-effective in 3D printing as they require less material when compared to regular lattice structures while supporting equivalent weight loads.^22^ Their customizable pore diameters and variable porosities also make 3D Voronoi structures highly appealing porous structures for modelling various materials and environments.^25^ The topological similarities may be defined in terms of structural parameters, such as the number of vertices (*V*), edges (*E*) or average edge length (*ε*). Unfortunately, these structural parameters have hitherto been unpredictable when designing a 3D Voronoi structure, thus requiring multiple iterations of differing design features, such as the number of generating points (*G*), to achieve a desired structural parameter (Fig. 1). Therefore, there is a need for data and equations relating the design features to the structural parameters, to design Voronoi structures more efficiently.

There have been various attempts to provide these data. However, these were often considering 2D Voronoi diagrams,^30–32^ single Voronoi cells,^33^ a small selection of possible design features,^34,35^ a highly specific type of Voronoi structure,^36^ or Voronoi scaffolds (which required a more complex set of design features, such as the mesh thickness).^37^

Here, we report correlations based on 12 000 cubic Voronoi structures built from different arrangements of three design features, by examining correlations with four different structural parameters, namely *V*, *E*, *ε*, and Euler's characteristic (*χ*). For each structural parameter, a correlation is derived from the analysis of these 12 000 Voronoi structures that will allow future Voronoi design features (*e.g.*, *G*) to be accurately predicted from the desired structural parameter (*e.g.*, *E* or *ε*). Section 5 presents three biomedical case studies to demonstrate the broad applications and accuracy of these equations.

Fig. 1 summarises the objectives. Table 1 lists the nomenclature used throughout this paper.

**Table 1:** Nomenclature used throughout the text and in the equations

Abbreviation	Nomenclature
CVT	Centroidal Voronoi tessellation
*λ*	Lloyd iterations
*G*	Number of generating points
*V*	Number of vertices
*E*	Number of edges
*F*	Number of faces
*ε*	Average edge length
*χ*	Euler's characteristic
*L*	Length of the cubic design space

## 2 Design methods

**Fig. 2:**
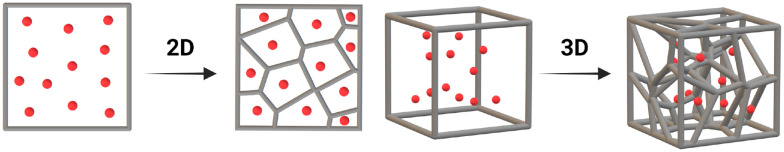
A 2D plane and 3D cube (left of the arrows) populated with 12 points. Those points are then used as the generating points to design a 2D Voronoi diagram and 3D Voronoi structure (right of the arrows).

Voronoi diagrams are designed by populating a defined space (such as a square or cube) with a certain number of points (*G*). Lines are then drawn equidistant to adjacent points resulting in a collection of Voronoi cells (Fig. 2).

**Fig. 3:**
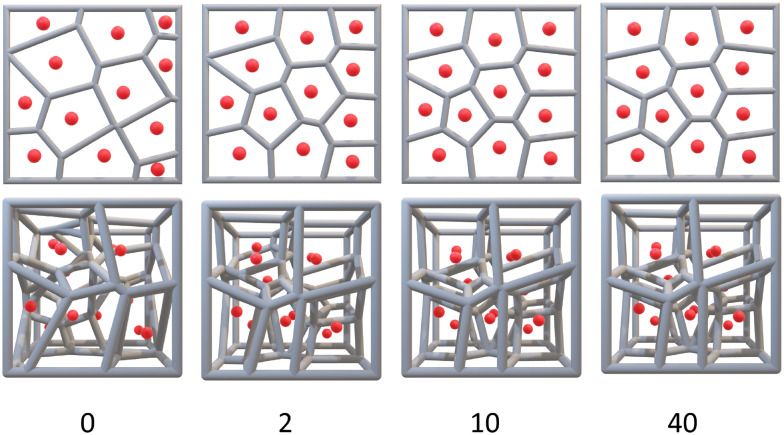
A collection of 2D Voronoi diagrams (top row) and 3D Voronoi structures (bottom row) after 0, 2, 10 and 40 Lloyd iterations. Both reach a centroidal Voronoi tessellation after 10–40 iterations. The 3D Voronoi structures are all Pose 5 and have an Euler's characteristic of 13. The first structure has 60 vertices, 116 edges and an average edge length of 32.74 units while the final three structures have 58 vertices, 112 edges and average edge lengths of 33.26, 33.12, and 33.11 units, respectively.

There are three common Voronoi design features: *G*, the orientation of those generating points in space (its Pose), and Lloyd iterations (*λ*).^38^ Lloyd iterations (named for its designer, Stuart P. Lloyd), iteratively average the area (volume) of each Voronoi cell within the 2D diagram (3D structure) and re-position the Voronoi generating points to the centroid of each cell.^39^ The Voronoi structure is then re-designed around the new generating points. Throughout a number of these iterations, a centroidal Voronoi tessellation (CVT) is achieved where no further iterations will shift the generating points (Fig. 3). As CVTs differ in various structural parameters when compared to non-CVTs (such as in stress-weight capabilities^23^), a range of 3D Voronoi structures with *λ* = 0–40 were designed.

Euler's characteristic (*χ*) was first presented by Leonhard Euler in 1758 where he noted common topological relationships between different 2D and 3D geometries.^40^ He presented this relationship for polyhedra in the form of eqn (1), where *χ* = 2:

1 *χ* = *V* − *E* + *F*

Different geometric types result in unique topological relationships (unique *χ*), including one for 3D polyhedra that contains a large aggregate of cells, such as a 3D Voronoi structure.^41^ This relationship is:

2 1 = *V* − *E* + *F* − Cells

where the number of “cells” would be the number of Voronoi generating points, resulting in:

3 *χ* = 1 + *G*

for a 3D Voronoi structure.

## 3 Computational methods

The Voronoi structures are designed in the visual programming language Grasshopper, within the Rhinoceros 3D™ software^42^ (for further information on Grasshopper, see the supplementary information, Section S3). For this study, the Voronoi boundary cube, within which the 3D Voronoi structures are designed, is set at *L* = 100 cm. This boundary cube may be scaled up or down, depending on the intended application.

**Fig. 4:**
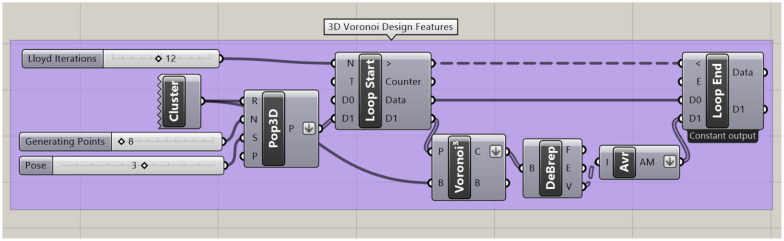
The Grasshopper code to design the 3D Voronoi structures and loop through the Lloyd iterations. The “cluster” component holds all the components required to build the initial *L* = 100 cm cube used as the Voronoi design space. The “Pop 3D” component then populates the boundary box with the defined number of points (set by the “generating points” number slider). The “Pose” slider indicates the saved orientation of those points in space. The “Voronoi^3^” component then generates the 3D Voronoi structure. The boundary representation (Brep) is then deconstructed with the “DeBrep” component before the “Avr” (average) operation is applied to locate each Voronoi cell centroid for examination. These centroids are then looped through the “loop end” back to the “loop start” component and set as the new generating points for the Voronoi structure. This continues for the number of iterations set in the “Lloyd iterations” slider.

To facilitate the Lloyd iterations, the loop component within the Grasshopper Anemone package is applied to iterate over the Voronoi cells' volumetric centres (Fig. 4).

**Fig. 5:**
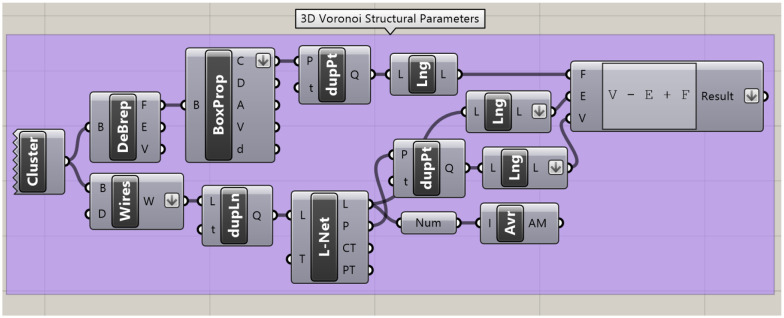
The Grasshopper code that calculates and provides the structural parameters for the 3D Voronoi structures. These parameters are the number of vertices, *V*; number of edges, *E*; average edge length, *ε*; and Euler's characteristic, *χ*. The “cluster” component holds all the components required to build the 3D Voronoi structure. The “DeBrep” (deconstruct Brep) and “Box Prop” (box properties) allow for each face within the Voronoi structure to be counted. Any duplicate points are removed through the “dupPt” (duplicate points) component and the number of faces is obtained through the “Lng” component (list length). This is introduced into Euler's equation (*V* − *E* + *F* = *χ*) along with the number of vertices and edges obtained through the “Wires” (wireframe) and “L-Net” (network from lines) components after removing any duplicate points (“dupPt”) and lines (“dupLn”). The average edge length is obtained from the “Avr” component, after converting the edge lengths from strings to numbers through “Num” component.

The four different structural parameters (*V*, *E*, *ε*, and *χ*) are calculated for each Voronoi structure. All parameters are determined using standardised Rhino components after all duplicate lines and points are removed, as shown in Fig. 5. The Poses were generated through a random number generation algorithm within Rhino.

This design code is then written into the Python plugin within Grasshopper and parallelized to increase the efficiency two–three fold.^14^ Using this code, thousands of Voronoi structures are generated in under a few hours. This open-access design code is provided in the supplementary information and allows any of over 12 000 structures to be downloaded as a 3D printable STL file. The code is fully adaptable to create 3D Voronoi structures of different shapes and sizes.

For this research, two sets of 3D cubic Voronoi structures are designed and examined to observe both the depth and breadth of different Voronoi patterns. Set A contains structures designed from *G* = 5–18, 20 Poses, and *λ* = 40 resulting in 11 200 structures. Set A covers structures with *V* = 25–90 and *E* = 50–170. However, as previous research into 3D Voronoi applications required more complex structures, a further 1250 structures are designed from *G* = 30–300, covering structures with *V* = 180–1600 and *E* = 400–3200 (set B).^16^ As the structural parameters observed in set A are negligibly affected by differing Poses and reached a CVT after *λ* = 10–25, only 5 Poses and *λ* = 25 are used in set B. All final results provided in section 4 are a combination of all 12 000 structures produced including all Poses and Lloyd iterations.

## 4 Voronoi equations

Over 12 000 different Voronoi structures are designed ranging from *G* = 5–300. The correlations between the different design features of these structures (*G*, Poses and *λ*) and the various structural parameters (*V*, *E*, *ε*, and *χ*) are then examined. From these data, equations predicting *G* based on *V*, *E*, *ε*, and *χ* are extracted. Correlations between *χ* and design space size are also examined as a function of *V*, *E*, and *ε*.

The graphs that describe the correlations between a structural parameter and *G* include a combination of Voronoi structures that undergo no Lloyd iterations (*λ* = 0) to those that have 25–40. Separate graphs comparing the structural parameters of Voronoi structures that only undergo 0–5 iterations and graphs that only examine CVTs are also produced. However, these graphs are highly similar to those that examine all the Voronoi structures with all the equations correctly predicting *G* within ±3. Therefore, the graphs below include all Voronoi structures, regardless of *λ*.

As the Pose of the points is randomly assigned, there is no direct correlation between the Pose and any of the parameters listed above. Therefore, the following sections only discuss the relationships between the structural parameters, *G* and *λ*. All data pertaining to Poses is in the supplementary information.

### 4.1 Vertices and edges

**Fig. 6:**
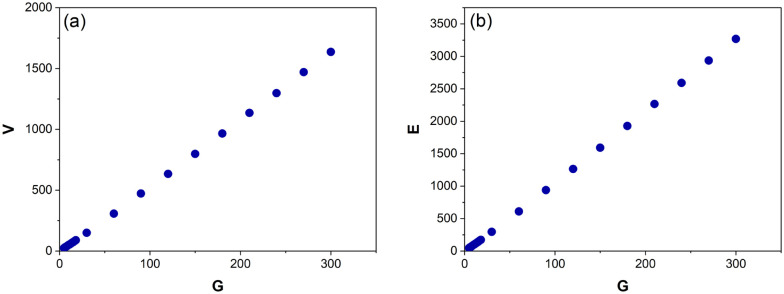
The average number of vertices, *V* (a) and edges, *E* (b) for Voronoi structures designed from 5–300 generating points (*G*). Each data point is an average of 125–800 Voronoi structures.

As shown in Fig. 6, *V* and *E* are directly proportional *G* (*R*^2^ = 0.99).

Performing a linear regression on these data above produces the following correlations both of which accurately predict *G* within ±1:

4 *G* = (*V* + 8.53)/5.45

5 *G* = (*E* + 21.10)/10.89

**Fig. 7:**
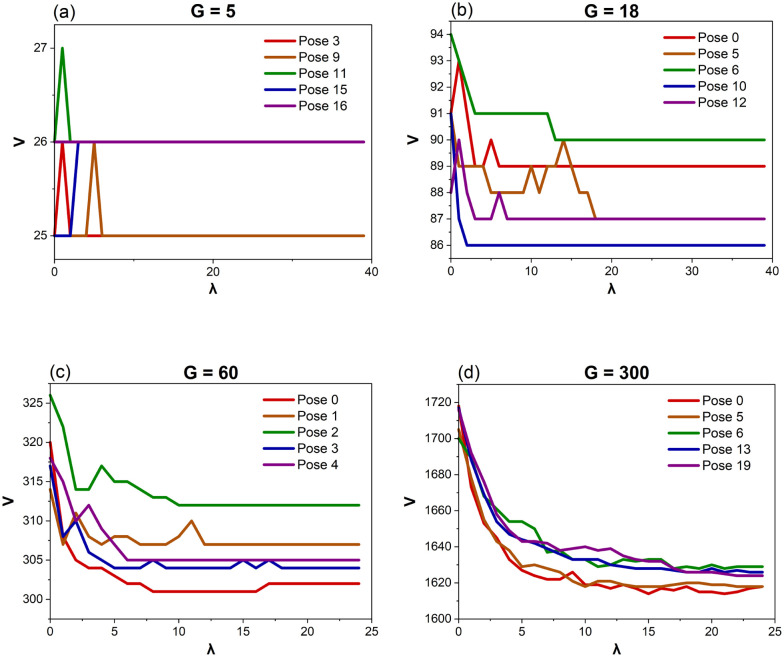
The average number of vertices (*V*) for each Lloyd iteration (*λ*) for Voronoi structures designed from 5 (a), 18 (b), 60 (c) and 300 (d) generating points (*G*). A selection of five configurations of the generating points (Poses) have been chosen for each *G*. This demonstrates the variety of different shifts within *V* due to the *λ*.

Graphs showing how *V* and *E* evolve as a function of *λ* are also examined. The final Voronoi structures (the one with the highest *λ*) have a lower *V* and *E* than the original Voronoi structure (*λ* = 0) in 172 of the 280 Poses (61%) generated from *G* = 5–18 (Fig. 7a and b). This is even more prominently observed in the structures designed with *G* = 30–300, where 49 of the 50 Poses (98%) have a lower *V* and *E* in the final Voronoi structure when compared to the first (Fig. 7c and d). This confirms that the Lloyd iterations are producing a CVT, which decreases the overall number of Voronoi cells in a structure, resulting in smaller *V* and *E* (Fig. 3). As *V* and *E* are directly proportional to each other (eqn (4) and (5)), only graphs pertaining to *V* are provided in Fig. 7.

For Voronoi structures with the same *G* and Pose, if a vertex is removed or added during a Lloyd iteration, this results in a corresponding removal or addition of two edges. This is expected, as Voronoi vertices are connected to four other vertices, except for the corner vertices. Therefore, the removal of one vertex leaves four vertices unconnected. The removal of two edges then allows the four vertices to be reconnected to each other with the remaining two edges. Corner vertices are only connected to three other vertices. If a corner vertex is removed, one of those two unconnected vertices now becomes a corner (only needing the remaining 3 edges). Therefore, two edges are still required to be removed to result in a fully connected Voronoi structure.

Out of the 11 200 structures designed from *G* = 5–18, only six (0.054%) deviate from this trend. Upon closer inspection, these six structures contain edges shorter than 0.015 cm, which results in a missing face or a duplicate vertex within the structure. Therefore, more or less than two edges are required to change if a vertex connected to that small edge is added or removed. However, there were 10 other structures with edges shorter than 0.015 cm that were consistent with the trend. Therefore, there may be additional characteristics that determine which edges result in missing faces or duplicate vertices.

As the Voronoi structures become more complex (structures generated from *G* = 30–300), a higher fraction of structures deviates from having the removal or addition of a vertex correspond to the removal or addition of precisely two edges during a Lloyd iteration (97 out of 1250 structures or 7.8%). All of these structures have edges shorter than 0.015 cm, which appear to show a physical limitation within Rhino as the edge lengths drop below 0.015 cm. However, due to the size and complexity of the structures designed from *G* = 30–300, it is challenging to confirm exactly which edge is causing the missing face or duplicate vertex. Nevertheless, 167 other structures, containing edges shorter than 0.015 cm, did follow the trend.

Theoretically, if *G* remains constant, the size of the cubic design space has no effect on *V* and *E*, as the design space is simply scaled up or down. However, to provide robust confirmation that eqn (4) and (5) can be applied to any cubic design space, over 4000 new Voronoi structures are designed (from *G* = 5–300) in different cube sizes (ranging from *L* = 10–150 units). All these structures have the same *V* and *E* as their respective Voronoi structures designed in the *L* = 100 cm design space. This confirms that both eqn (4) and (5) can be applied to any cubic Voronoi structure.

### 4.2 Average edge length

**Fig. 8:**
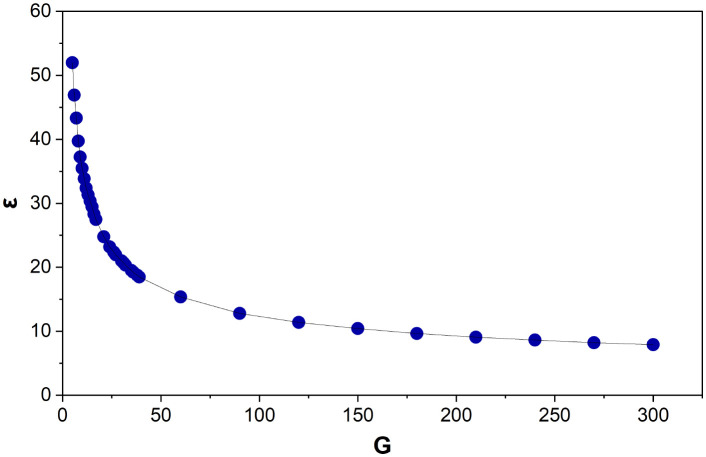
The average, of the average edge lengths (*ε*) for Voronoi structures designed from 5–300 generating points (*G*). Each data point is an average of 125–800 Voronoi structures.

A power law relationship (*R*^2^ = 0.99) is observed between *G* and *ε* (Fig. 8):

6 *G* = (*ε*/101.41)^(−1/0.45)^

When tested, this equation accurately predicts *G* within 0–10, with an average deviation of ±3.

Unlike *V* and *E*, *ε* depends on the size of the cubic design space the Voronoi structure is built in. For *G* to be dimensionless the denominator, 101.41, needs to be in the same units as *ε*. As the cube is designed from *L* = 100 cm (very close to 101.41), this provides additional confirmation to the accuracy of this equation. Therefore, it is proposed to simplify this to:

7 *G* = (*ε*/*L*)^(−1/0.45)^

where *L* is the length of the cubic design space. This equation accurately predicts *G* within 0–11 (average deviation ±3). Although the deviation is slightly higher than for eqn (6), this equation proves highly accurate for changing box sizes, as discussed below.

Over 4000 structures designed in different cube sizes (*L* = 10–150 units) conform to eqn (7). The deviation is 0–5 (average ±2) between the calculated and known value of *G*. This is an even higher level of accuracy compared to eqn (7)'s prediction for the original set of Voronoi structures.

Data correlating *ε* and *λ* are also examined. The Voronoi structures designed from *G* = 5–60 show no further fluctuations in *ε* after *λ* = 15–20, indicating a CVT is reached. However, those designed from *G* = 90–300 continue to show slight fluctuations in *ε* even after *λ* = 25.

### 4.3 Euler's characteristic

**Fig. 9:**
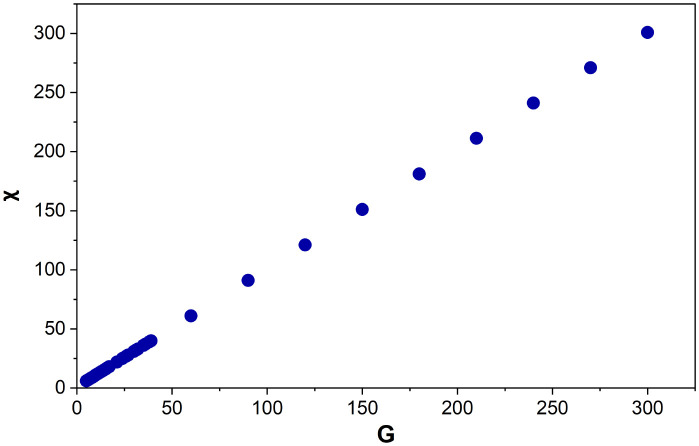
Euler's characteristic (*χ*) for Voronoi structures designed from 5–300 generating points (*G*). Each data point is an average of 125–800 Voronoi structures.

There is a linear relationship (*R*^2^ = 1) between *G* and *χ* (Fig. 9).

Performing a linear regression on the data above produces eqn (3). This demonstrates that the Voronoi structures designed, accurately follow Euler's eqn (3) for 3D polyhedra. The only structures that deviate from eqn (3), are mostly the same ones mentioned in section 4.1, which were missing a face or contained a duplicate vertex. There are 13 additional structures (designed from *G* = 210–300), where the addition and removal of a vertex corresponds to two edges but the Euler values deviate from eqn (3). This appears to show that multiple missing faces or duplicate vertices have occurred in these structures which, together, still result in the addition and removal of vertices corresponding to two edges but not Euler's characteristic conforming to eqn (3).

**Fig. 10:**
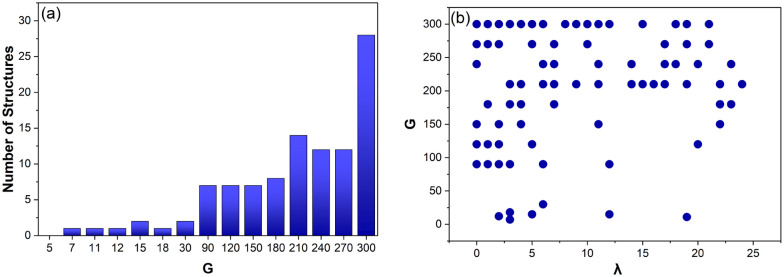
The number of Voronoi structures designed from a specific number of generating points (*G*) that have an Euler characteristic, not equal to 1 + *G* (a). These same structures are then ranked according to the number of Lloyd iterations (*λ*) they have completed (b).

Fig. 10a shows how the number of structures that deviate from eqn (3) increases as the complexity of the structures increases (fewer edges shorter than 0.015 cm). These structures are also compared with *λ* (Fig. 10b). This shows that 76% (78/103) of all these structures occur for *λ* < 15, indicating that the closer the structure is to a CVT, the less likely it is to have an edge shorter than 0.015 cm and deviate from eqn (3). This also confirms that Lloyd iterations are producing CVT that decrease the overall number of Voronoi cells in a structure, reducing the occurrence of shorter edges.

### 4.4 Summary table

Table 2 contains the full toolkit of the equations presented in this paper in order to design Voronoi structures purely off a desired structural parameter.

**Table 2:** A list of equations that predict the number of Voronoi generating points (*G*) based on a specific structural parameter. The respective ranges of the various structural parameters the equation has been tested in, and the average deviation is also provided. The number of vertices, edges, average edge length, the length of the Voronoi cubic design space, and Euler's characteristic are represented as *V*, *E*, *ε*, *L*, and *χ*, respectively

	Equation	Range tested	Average deviation
(4)	*G* = (*V* + 8.53)/5.45	*V* = 25–1700	±1
(5)	*G* = (*E* + 21.10)/10.89	*E* = 45–3400	±1
(7)	*G* = (*ε*/*L*)^(−1/0.45)^	*ε* = 7–55	±3
(3)	*G* = *χ* − 1	*χ* = 6–301	±0

## 5 Biomedical applications

Voronoi tessellation is widely used in the modelling of biological structures.^15,16^ When applied to disease modelling, Voronoi structures could enable *in vitro* experimentation, as well as *in silico* simulation of complex microenvironments. The equations presented in Table 2 provide a practical toolkit allowing Voronoi structures to be efficiently built based on the desired values for *V*, *E*, *ε*, and *χ*. These equations are then tested in a collection of different biomedical case studies to determine their accuracy and effectiveness.

Firstly, eqn (4) is tested in the design of a 3D T-cell culturing structure, where the Voronoi structure is modelled off the fibroblastic reticular cell (FRC) networks of the lymph nodes.^15^ Previous research has shown that, based on FRC networks in the T-cell zone of a mouse lymph node, approximately 65 vertices fit inside a *L* = 100 μm cube for a Voronoi structure design.^43^ Inserting this desired parameter into eqn (4) produces *G* = 13 and 800 new structures are then designed from these generating points to test how accurately eqn (4) performed. Of the 800 structures, 95 have exactly 65 vertices and the other ones have between 61 and 68. To compare this with structures designed from *G* = 12 and 14, 800 new structures are also generated from those generating points. None of the structures generated from *G* = 12 or 14 have 65 vertices (Fig. 12). This demonstrates that eqn (4) has correctly predicted the number of generating points required to achieve the desired structural parameter of 65 vertices.

Secondly, eqn (5) is tested in the design for a 3D Voronoi structure of a patient's trabecular bone, which may be used to examine the structural fragility resulting from osteoporosis.^44^ As Voronoi structures have been shown to accurately model the bone microenvironment,^16,24^ a Voronoi structure is designed to model a patient's trabecular bone derived from *E* calculated in a segment of the patient's bone microenvironment (1388 edges).^44^ However, a more detailed model would require further parameters, such as anisotropy and inhomogeneity, to increase accuracy. Inserting this value into eqn (5), produces the estimated value of *G* = 129. To test the accuracy of the predicted value, 100 Voronoi structures are designed from 129 generating points. The 100 structures have a range of *E* = 1342–1438 with an average of 1376 edges. As with the T-cell culturing environment, structures with *G* on either side of the predicted value (128, 130 and 131) are also examined. The 50 structures designed from *G* = 128 have an average of 1363 edges, *E* = 1386 for those generated from *G* = 130 and *E* = 1399 from *G* = 131 (Fig. 12). It is noted that the structures generated from *G* = 130 have an average number of edges closer to the desired value of 1388 than those generated from 129 points. However, the prediction from eqn (5) is still within the listed deviation of *G* ± 1 (Table 2).

Thirdly, eqn (7) is tested in the design of a 3D Voronoi structure of a kidney cancer tumour, which can be used to increase a patient's understanding of a complex ontological surgical procedure or provide a surgical team with an in-depth model of the impending operation.^26,27^ Saribudak *et al.*^13^ designed a 2D Voronoi model from a kidney cancer tumour with *ε* = 35.0 mm (measured using hematoxylin–eosin staining).^13^ Although this parameter was for a 2D Voronoi diagram, it is applied to a 3D Voronoi structure to demonstrate the accuracy of eqn (7). For a complete and accurate model of a kidney cancer tumour, a 3D histological image of the cell–cell connections within the tumour would need to be examined. The *ε* of 35.0 mm is set as the desired structural parameter for a 3D Voronoi structure built in a *L* = 150 mm design space. These parameters are then inserted into eqn (7) where the predicted result is *G* = 25. Therefore, 100 Voronoi structures are designed from 25 points and *ε* is calculated to be 34.1 mm. Once again, 100 Voronoi structures are also designed from *G* on either side of the predicted range (23, 24 and 26). Those generated from 23 lead to *ε* = 35.5 mm; for those from *G* = 24, *ε* = 34.8 mm and for those from *G* = 26, *ε* = 33.6 mm (Fig. 12). It is noted that the structures generated from *G* = 23 and 24 have an *ε* closer to the desired value of 35.0 mm than those generated from 25. However, the prediction from eqn (7) is still within the listed deviation of *G* ± 3 (Table 2).

These equations also provide a high level of efficiency in predicting the required parameters to design 3D Voronoi structures. In Rhino a single 3D Voronoi design code may take 30 s to design each structure, including time for changing parameters. Reducing the number of structures required to achieve specific design parameters from dozens or hundreds to two or three, results in computational times being reduced from 30–50 min to 1–2 min.

**Fig. 11:**
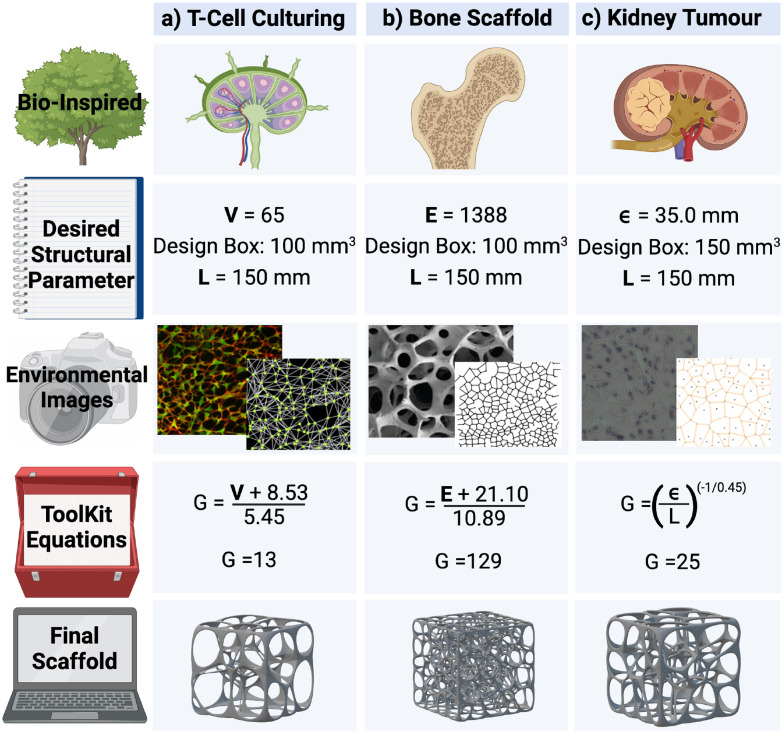
A visual representation of three different biomedical applications of 3D Voronoi structures, where the Voronoi design features (generating points – *G*) have been calculated purely off desired structural parameters (number of vertices – *V*, edges – *E* and average edge length – *ε*). The edge length of the cubic design space has been abbreviated to *L*. The images of the 3D Voronoi structures have been converted to scaffolds (had a mesh wrapped around them) for illustration purposes. The environmental images displayed are from the following papers, for the lymph nodes (a),^43^ bone (b)^17,44^ and kidney tumour (c).^13^

These three case studies have been visually represented in Fig. 11.

**Fig. 12:**
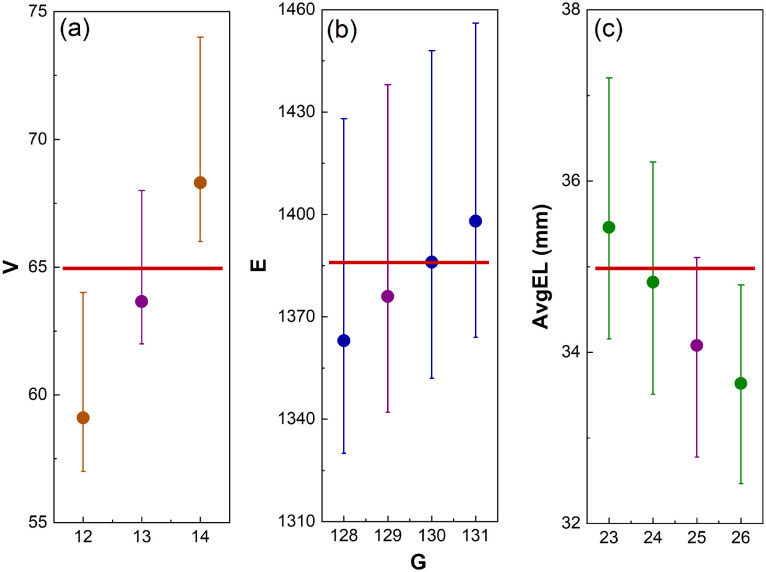
The accuracy of the presented equations to predict the number of generating points (*G*) required to achieve a desired structural parameter. Three different structural parameters are examined: (a) the number of vertices – *V*, (b) the number of edges – *E*, and (c) the average edge length – *ε*. The predicted *G* has been shown in purple with the desired structural parameter demonstrated as a red line (*V* = 65, *E* = 1388, and *ε* = 35 mm). The error bars demonstrate the maximum and minimum structural parameter values obtained.

## 6 Conclusion

This research provides a toolkit of equations, based on over 12 000 3D Voronoi structures, that allows a 3D Voronoi number of generating points (*G*) to be efficiently and accurately predicted based on the desired structural parameters (number of vertices – *V*, edges – *E*, average edge length – *ε*, and Euler's characteristic – *χ*). With these equations, we propose, to the best of our knowledge, two new mathematical conjectures that relate the *V*, *E*, and *ε* to *G* in a Voronoi contained in a cube. These equations have been validated for a wide range of parameter values (*V* = 25–1700, *E* = 45–3400, *ε* = 7–55 cm, and *χ* = 6–301) for different Voronoi network sizes (*L* = 10–150 cm). They are shown to accurately predict the number of generating points required to model a lymph node based on the number of vertices, to model a trabecular bone based on the number of edges, and to model a kidney tumour based on the average edge length all within *G* ± 3. The observed linear relationships between *G* and *V* (or *E*) are robust across various Lloyd iterations.

In the future, structures could be generated under other boundary conditions (*e.g.*, beyond cubes) to examine the broader applicability of these correlations. Nevertheless, further mathematical analysis would be required to examine whether the empirical relationships described in this work are fundamental. Moreover, this toolkit may be expanded to include equations that would predict design features for Voronoi scaffolds (structures with a mesh wrapped around them), such as porosity, surface area or curvature. Although this paper has chosen to focus on biomedical applications, these equations would hold the same level of accuracy for modelling any of the environments listed in the introduction. The 3D Voronoi design code, written in the Grasshopper application within Rhino 3D™,^42^ has been provided allowing any of the thousands of structures to be selected, easily adjusted based on user requirements, and 3D printed for the wide variety of ever developing applications.

## Data availability

Supplementary information for this article is available at DOI: https://doi.org/10.17632/bvxrgng7y2.1.

## Author contributions

Lucy Todd: conceptualization (lead), methodology (lead), software, validation, formal analysis, investigation, data curation, writing – original draft, visualization. Matthew H. W. Chin: conceptualization (equal), methodology (equal), resources, supervision, writing – review & editing. Marc-Olivier Coppens: conceptualization (equal), methodology (equal), writing – review & editing, supervision, funding acquisition.

## Conflicts of interest

The authors have no conflicts to declare.
